# Presentation of lung cancer in primary care

**DOI:** 10.1038/s41533-019-0133-y

**Published:** 2019-05-22

**Authors:** D. P. Weller, M. D. Peake, J. K. Field

**Affiliations:** 1 Usher Institute, University of Edinburgh, Edinburgh, UK; 2 Centre for Cancer Outcomes, University College London Hospitals Cancer Collaborative, University of Leicester, NCRAS/PHE, London, UK; 3 Roy Castle Lung Cancer Research Programme, Department of Molecular and Clinical Cancer Medicine, The University of Liverpool, Liverpool, UK

**Keywords:** Respiratory signs and symptoms, Signs and symptoms

## Abstract

Survival from lung cancer has seen only modest improvements in recent decades. Poor outcomes are linked to late presentation, yet early diagnosis can be challenging as lung cancer symptoms are common and non-specific. In this paper, we examine how lung cancer presents in primary care and review roles for primary care in reducing the burden from this disease. Reducing rates of smoking remains, by far, the key strategy, but primary care practitioners (PCPs) should also be pro-active in raising awareness of symptoms, ensuring lung cancer risk data are collected accurately and encouraging reluctant patients to present. PCPs should engage in service re-design and identify more streamlined diagnostic pathways—and more readily incorporate decision support into their consulting, based on validated lung cancer risk models. Finally, PCPs should ensure they are central to recruitment in future lung cancer screening programmes—they are uniquely placed to ensure the right people are targeted for risk-based screening programmes. We are now in an era where treatments can make a real difference in early-stage lung tumours, and genuine progress is being made in this devastating illness—full engagement of primary care is vital in effecting these improvements in outcomes.

## Introduction

Lung cancer poses a significant public health burden around the world; it is the most common cause of cancer mortality in the UK and it accounts for >20% of cancer deaths.^1^ There is significant variation in survival rates around the world and this has been largely attributed to the stage at which the cancer is diagnosed.^2^ The International Cancer Benchmarking Partnership has demonstrated that survival rates in the UK lag behind those of other countries, and late diagnosis is thought to be a major underlying factor.^3,4^ Importantly, patients with early-stage disease have a much better prognosis; stage 1 non-small-cell lung cancer can have a 5-year survival rate as high as 75%.^5^ Even within the UK, however, there is wide variation in lung cancer survival rates and in the proportion of patients diagnosed with early-stage disease.^6^

In the UK, most cancers present symptomatically in primary care (most commonly to a general practitioner, or ‘GP’, the medical lead of a primary care team), and the diagnosis is made after a referral for either investigations or directly to secondary care.^7^ Many of the symptoms of lung cancer are very common but non-specific in primary care practice: these include chest pain, cough and breathlessness;^8^ hence, lung cancer poses a very significant diagnostic challenge—a primary care practitioner (PCP) working full time is likely to only diagnose 1 or 2 cases per year. Further, lung cancer often emerges on a background of chronic respiratory disease and symptoms of chronic cough—typically in patients who smoke. It can be very difficult to identify changes in these chronic symptoms that might indicate the development of a lung tumour.

Smoking remains the principal aetiological factor and smoking cessation is the key public health initiative to reduce mortality from this disease;^9^ indeed, at almost any age smoking cessation can produce health benefits. Hence, public health campaigns to promote smoking cessation, supplemented by strategies in primary care based on nicotine replacement therapies should be encouraged.^10^ The role of e-cigarettes is not yet fully understood,^11^ although any strategy that reduces exposure to tobacco smoke has a potential for producing significant benefits.

## How do patients respond to lung cancer symptoms?

There is a significant body of research around patient response to symptoms that might potentially indicate lung cancer. Because symptoms often present within the context of chronic respiratory symptomatology, changes associated with the development of a tumour may go un-noticed or be dismissed.^8^ It is known that patients often delay their help seeking through a range of psychological mechanisms including denial and nihilism—hence, there can often be significant delays before patients present to primary care.^12,13^

There is evidence for variation in the timeliness of presentation of lung cancer in between countries; people with lung cancer often have symptoms for a considerable period of time before they present to primary care and this is a major source of delay in the diagnostic process with potential adverse impact on survival;^14,15^ this patient interval does, however, vary between studies. It is important that PCPs understand some of the psychological mechanisms that either promote or inhibit early presentation among their patients.

## Public awareness of lung cancer

**Fig. 1:**
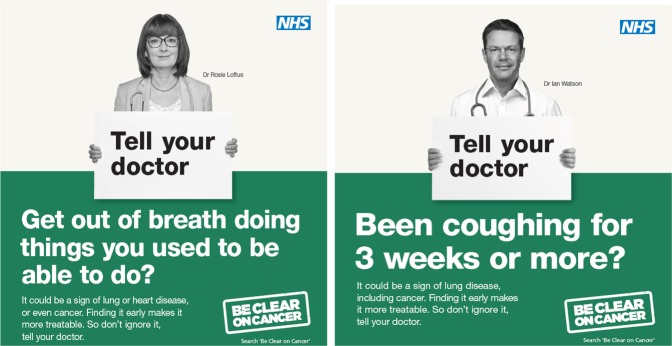
Posters used in the ‘Be Clear on Cancer’ campaign

Over the past few years, there have been campaigns run throughout the UK designed to make the public more aware of symptoms associated with lung cancer—for example the ‘Be clear on Cancer’ campaign run by Public Health England and ‘Diagnose Cancer Early’ in Scotland^16,17^ (see Fig. 1). These campaigns have demonstrated an ability to diagnose additional cancers and effect modest increases in the proportion of patients having tumours diagnosed at stages where they are amenable to resection.^18,19^

Of course, lung cancer early detection programmes need to be focussed on the hard-to-reach population and those who will benefit most from involvement; there are often concerns expressed over burdening services with patients with insignificant symptoms^18^ and an emerging consensus that all stakeholders should be closely engaged in the campaigns. Nevertheless, available evidence suggests that lung cancer could be diagnosed earlier through these public awareness campaigns,^19^ particularly when associated with systems to help primary care physicians risk stratify their patients for lung cancer more effectively—indeed, further work to identify patients who might benefit from targeted interventions should be a priority.

Community-based social marketing interventions have a potential key role;^20^ they can increase the likelihood of patients attending PCPs and increase primary care diagnostic activity (such as chest X-ray referrals)—as well as increases in lung cancer diagnostic rates. The level of suspicion at which PCPs consider a referral is a key factor in response to these campaigns—and there are concerns over ‘system overload’ through encouragement to present with symptoms.^13^ Ideally, campaigns might preferentially target those at greater risk of lung cancer, such as people with significant smoking histories or occupational exposure.

## Primary care response to lung cancer symptoms

In the UK, GPs will on average only diagnose one or two cases of lung cancer per year (if they are in full-time practice).^21^ However, during that year, GPs will see hundreds of patients with common symptoms, such as cough, breathlessness and chest pain—hence, there are significant difficulties in identifying, diagnosing and referring these patients in a timely manner.

The 2015 NICE lung cancer guidelines on recognition and referral^22^ have underpinned some important strategies to enhance timely lung cancer diagnosis; in many regions of the UK, there are now accelerated diagnostic pathways that assist GPs in identifying and referring patients appropriately.^23^ Audit data demonstrate that there are typically several consultations prior to a diagnosis of lung cancer being made.^24^ Evidence from significant event analysis in the UK has suggested that there is timely recognition and referral of symptoms in primary care;^25^ longer intervals are typically attributed to factors such as X-rays being reported as normal, patient-mediated factors and presentations complicated by co-morbidity. The importance of safety netting has also been emphasised in presentations where a diagnosis of lung cancer is possible.^26^

There needs to be continued work to counteract the ‘nihilism’ associated with lung cancer; PCPs are very well aware of patients who may suspect they have lung cancer but fail to present either because they blame themselves (through a history of smoking) or because they believe that if a cancer is diagnosed there is little that can be done about it.^27^ This, coupled with the tendency for patients in the UK to be concerned about ‘bothering the doctor’,^28^ can have detrimental effects on early diagnosis.

While public campaigns can do much to overcome barriers to presentation, it is vital that PCPs become more pro-active in achieving more timely diagnosis in their practice populations. It is been recommended that they should recognise the psychological mechanisms that might underlie patient delay and tackle nihilistic attitudes through educational and motivational strategies.^29^ Indeed, there is cause for cautious optimism with new treatments, and this should be conveyed to patients; for example, the use of stereotactic radiotherapy and volume-sparing surgery means that patients who previously could not be offered curative treatment due to co-morbidities are often now eligible.^30^

Audits that systematically identify at-risk patients who may be failing to present are a potential way forward; interventions which identify and target high-risk patients appear feasible in primary care.^31^ Crucially, patients should be reassured that PCPs are always happy to see them if they are worried about potential cancer symptoms.

## Risk assessment and lung cancer

It is vital in assessing lung cancer risk to look carefully at lifestyle factors and past medical history; only one in seven cases of lung cancer occur in people who have never smoked, and the presence of chronic obstructive pulmonary disease doubles the risk independent of smoking history.^32^ A previous history of head and neck, bladder and renal cancers and other factors such as exposure to asbestos or living in high radon exposure areas are all important in lung cancer risk assessment. Family history produces an excess of risk and should be included in risk assessment—as should the symptom of fatigue, a common feature of lung cancer. Cancer decision support tools such as the ‘Caper’ instrument or ‘Q cancer’ have emerged in recent years in the UK, enabling GPs to make assessments of cancer risk based on presenting symptoms;^33,34^ they have been incorporated into clinical systems in primary care with mixed results.

Beyond these symptom-based models, a number of lung cancer risk models have been developed based on validated epidemiological criteria—for example, the Liverpool Lung Project (LLP) risk model^35^ (www.MyLungRisk.org), which was subsequently used in the UK Lung Cancer Screening Trial.^36^ The LLP_v2_ risk model has also been used in the Liverpool Healthy Lung project,^37^ which has accommodated the risk model within primary care practice and produced risk assessments that are useful in clinical decision making is now running into its third year. The Manchester lung cancer pilot study^38^ has used the PLCO_2012_ risk prediction model^39^ and the recent Yorkshire Lung cancer screening trial^40^ is using both the LLP_v2_ and the PLCO_2012_ risk models. Models such as these provide a systematic way of assessing lung cancer risk, taking into account a range of factors, including smoking duration, previous respiratory disease, family history of lung cancer, age, previous history of malignancy and asbestos exposure.

Risk stratification in primary care is clearly a key priority. We need to look at instruments such as the LLP model and identify ways that lung cancer risk stratification can be made easy and convenient in primary care. At present, it is not possible to recommend a specific risk assessment tool for use in primary care; current ongoing research in primary care is externally validating existing tools and will compare their efficacy.^41^ Acceptability and feasibility also need to be examined; complex algorithms that place extra burden on practitioners are unlikely to succeed. However, we do need to ensure that the basic risk prediction parameters are correctly documented in primary care, so they can be utilised in any future national lung cancer screening programme approved by the UKNSC. We also need a better understanding of ways to maximise benefits of these models—while minimising potential harms such as over-medicalisation, anxiety and false reassurance.^42^ Machine learning or neuro-linguistic programming, whereby data from multiple practice-based and external sources might be examined to develop risk estimates, are also likely to play a significant role in the future.^43^

## Diagnostic pathways

Early diagnosis lung cancer clinics based on multi-disciplinary teams (MDTs) are an ideal option for expediting diagnosis—ideally with an urgent (2-week wait) referral;^44^ there is good evidence that these specialist MDT clinics are associated with improved outcomes. Another important consideration is involving the whole primary care team and including other practitioners such as pharmacists who see a lot of patients with, for example, repeat purchases of cough medicine. There has been a push to change referral practices in some parts of the UK—for example, to lower the threshold that PCPs refer for chest X-ray^45^ and to encourage practitioners to repeat the investigation after a few months if symptoms persist; critically a normal chest X-ray does not exclude diagnosis of lung cancer. One highly successful programme in Leeds included the option for people to self-refer for chest X-rays in walk-in clinics^19^—a crucial element was the engagement of primary care in the design and implementation of the programme.

Diagnostic pathways have been closely examined and tested over recent years, an example being CRUK’s ACE programme (accelerate, coordinate and evaluate) initiated in June 2014 in England and Wales.^23^ Patients often have complex pathways that can lead to delays; important initiatives in the ACE programme and elsewhere include risk-stratified computed tomographic (CT) screening criteria for ‘straight to CT’ referrals following normal chest X-rays and a focus on diagnostic paths for patients with vague symptoms.

Work needs to continue on diagnostic pathways that might expedite lung cancer diagnosis. It is important, for example, that we get more evidence on the impact or potential impact of direct access to investigations such as spiral CT from primary care—at present, there is not sufficient evidence or resource to universally implement this strategy, and there is evidence that delays can occur in primary care (for example, through ordering too many chest X-rays.^46^ Nevertheless, GPs in the UK often indicate that direct access to investigations would help streamline diagnosis.^7^

## Lung cancer screening

A major challenge for primary care is the lack of symptoms in very early stage lung cancer, highlighting the importance of examining the potential of screening. The US National Lung Cancer Screening Trial, which used low-dose CT scanning in high-risk patients, showed a 20% reduction in lung cancer-specific mortality and almost a 7% reduction in all-cause mortality—and the US Preventive Task Force on Lung cancer Screening recommended that lung cancer screening should be implemented in high-risk populations.^47,48^ Accordingly, Medicare agreed to pay for lung cancer screening within certain criteria—however, the current uptake in the US is only ~2% of high-risk individuals.

**Fig. 2:**
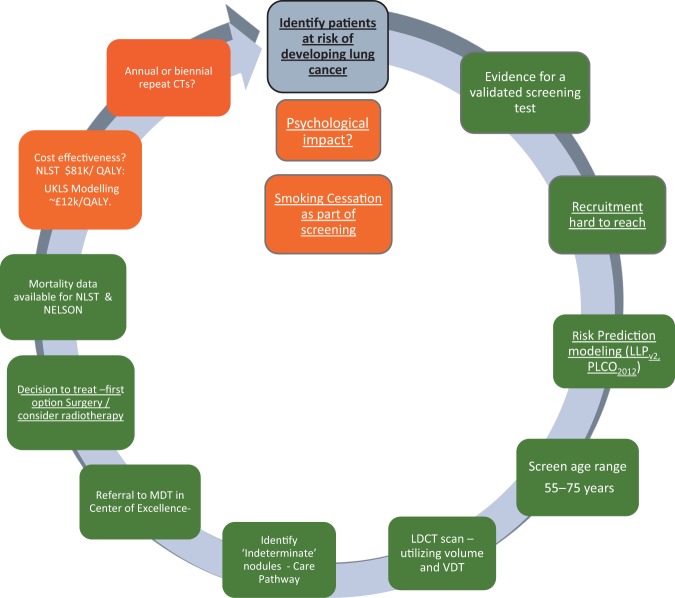
Levels of evidence for the implementation of lung cancer screening in Europe. The colour codes refer to the current status March 2019; traffic lights: green—ready, amber—borderline evidence. Underlined text indicates particular relevance for primary care^53^

The recent report on the NELSON trial at the World Lung Cancer Conference, Toronto^49^ has demonstrated an encouragingly low rate of false positives and a mortality benefit of 26% in men and between 39% and 61% in women—depending on the number of years of follow-up (i.e. 8–10 years). These results provide further impetus for the introduction of spiral CT scanning for individuals at high risk of cancer in the UK. Figure 2 illustrates the process for identifying an appropriate screening population, recruiting them and implementing screening—in many ways more complex than existing cancer screening programmes where recruitment is based principally on age and gender.

If we are, indeed, on the cusp of a new screening programme, there are important implications for primary care; the key issue in lung cancer screening is identifying the right patients to invite. This is a task that would involve primary care which currently lacks the systems and the processes to undertake the kind of population- based lung cancer risk assessment required. It is important, therefore, that we plan for an era where high-risk patients are screened for lung cancer (implemented, ideally, in tandem with smoking cessation programmes). We should be refining current strategies to risk stratify patients in primary care in preparation for this new era.^50,51^ Screening alone, however, is not the total answer and a high level of awareness in both the public and the primary care community will remain vital elements in what needs to be a multi-pronged approach.^52^

## Conclusions and recommendations

Mortality rates for lung cancer remain stubbornly high; if we are to improve lung cancer outcomes, it is important that early diagnosis and screening efforts achieve their maximum potential. We need to:

- identify ways of raising awareness of symptoms potentially associated with lung cancer in ways that encourage people at higher risk to come forward—this will require refinement of the messages delivered in awareness-raising strategies

- counter the nihilistic beliefs often associated with lung cancer—early diagnosis CAN lead to improved outcomes
- continually strive to improve the primary care response to patients with symptoms of lung cancer, supported by better diagnostic pathways and risk-based decision support
- identify ‘fail-safe’ mechanisms by which patients advised to ‘watch and wait’ are not lost to follow-up; it is vital that patients understand these safety netting and follow-up advice
- ensure that the basic risk prediction parameters are correctly documented in primary care, so they can be utilised in any future national lung cancer screening programme approved by the UKNSC
- refine methods to implement lung cancer risk assessment model approaches; this is key to improving diagnosis of early lung cancer—and we should aim for risk estimates that can be readily incorporated into the various kinds of practice software used in primary care practices
- continue to improve diagnostic pathways; at present, many different models are being evaluated, including those which give primary care more direct access to investigations such as spiral CT. The key task will be implementation and appropriate support once the best models are determined
- fully engage primary care with the likely implementation of spiral CT lung cancer screening in the next few years—this will require the best possible risk-stratification approaches to ensure screening is directed at those who stand to benefit the most from it. It is vital that primary care rises to this challenge

Primary care needs to play a central role in efforts to diagnose lung cancer earlier, if there is to be an improvement in lung cancer outcomes in the years ahead. Research over the past decade gives us a much clearer idea of what needs to be done in refining primary care-based strategies; with adequate commitment and resources primary care will, in conjunction with other health care sectors, help reduce the burden from this disease.
